# Three-dimensional visualisation of authentic cases in anatomy learning – An educational design study

**DOI:** 10.1186/s12909-022-03539-9

**Published:** 2022-06-20

**Authors:** Charlotte Silén, Klas Karlgren, Hans Hjelmqvist, Björn Meister, Hugo Zeberg, Anna Pettersson

**Affiliations:** 1 Department of Learning, Informatics, Management and Ethics, Karolinska Institutet, Stockholm, Sweden; 2 Department of Research, Education, Development and Innovation, Södersjukhuset, Stockholm, Sweden; 3 Faculty of Health and Social Sciences, Western Norway University of Applied Sciences, Bergen, Norway; 4 School of Medical Sciences, Örebro University, Örebro, Sweden; 5 Department of Neuroscience, Karolinska Institutet, Stockholm, Sweden; 6 Department of Evolutionary Genetics, Max Planck Institute for Evolutionary Anthropology, Leipzig, Germany; 7 Department of Neurobiology, Care Sciences, and Society, Division of Physiotherapy, Karolinska Institutet, Solna, Sweden

**Keywords:** Anatomy, Educational Design, Learning, Visualisation

## Abstract

**Background:**

Many studies have investigated the value of three-dimensional (3D) images in learning anatomy. However, there is a lack of knowledge about students learning processes using technology and 3D images. To understand how to facilitate and support the learning of anatomy, there is a need to know more about the student perspectives on how they can use and benefit from 3D images.

**Methods:**

This study used designed educational sessions informed by Educational Design Research to investigate the role of technology-enhanced 3D images in students’ anatomy learning. Twenty-four students representing different health professions and multiple study levels, and one tutor, participated in the study. A visualisation table was used to display the images of real patient cases related to disorders associated with the abdomen and the brain. Students were asked to explore the images on their own and audio/video capture was used to record their words and actions. Directly following the session, students were interviewed about their perceptions and different ways of learning and studying anatomy. The tutor was interviewed about his reflections on the session and his role as a facilitator on two occasions. Content analysis was used in its manifest and latent form in the data analysis.

**Result:**

Two main categories describing the students’ and tutor’s accounts of learning using the visualisation table were identified: 1. Interpreting 3D images and 2. Educational sessions using visualisation tables. Each category had signifying themes representing interpretations of the latent meaning of the students' and tutor's accounts. These were: Realism and complexity; Processes of discernment; References to previous knowledge; Exploring on one's own is valuable; Context enhances learning experiences; Combinations of learning resources are needed and Working together affects the dynamics.

**Conclusions:**

This study identifies several important factors to be considered when designing effective and rewarding educational sessions using a visualization table and 3D images in anatomy education. Visualisation of authentic images has the potential to create interest and meaningfulness in studying anatomy. Students need time to actively explore images but also get tutor guidance to understand. Also, a combination of different resources comprises a more helpful whole than a single learning resource.

## Background

The growing interest in technology-enhanced learning has involved an intense development of various software programs to support anatomy education, not least technology that can display digital three-dimensional views of anatomical features. There are different terms in the literature to describe this technology. In this article the term 3D images will be used for computed tomography scans (CT) and magnetic resonance imaging (MRI) which are displayed as three-dimensional (3D) images. This is reflected in research with numerous studies comparing the effectiveness of different formats of 3D images to other methods of teaching anatomy such as dissections, lectures, 2D images, living anatomy courses, plastination, and combinations of a range of approaches [1, 2]. The usefulness of 3D images is confirmed in several literature reviews [1, 3–6]. A challenge when designing learning activities is to understand how students interpret 3D images. Making sense of anatomical 3D images builds on the ability to discern crucial structures and combine them into an understanding of what you are looking at. Once you can discern certain anatomical structures and identify what you see, they cannot be unnoticed [7]. Therefore, experts, such as anatomy teachers, will interpret images differently and more accurately than students do. Consequently, an expert may no longer understand what it is like to be a novice looking at the same images. So how will the knowledgeable teacher understand what a student discerns when looking at 3D images in the learning of anatomy? Designing appropriate and well-thought-through learning activities involving display of 3D images requires understanding student perspectives and perceptions. To complement student perceptions, there is also a need to understand the role and perceptions of the teacher in supporting students learning anatomy.

### Distinguishing the types of 3D resources

Different technologies can be used to achieve a sense of depth and three-dimensionality in anatomical visualisations. Conventional computers screens can make use of the depth cues in images such as interposition, occlusion, size, shading, surface texture gradients, and brightness [5]. Hackett and Proctor refer to this method as ’monoscopic 3D' although it may occasionally also be referred to as "2.5D" or "pseudo-3D". Another method is to deliver two different views of an image ("stereo pairs") to the viewer who with the help of 3D glasses gets binocular depth cues through the combined visual information from slightly different images (stereopsis). Hackett and Proctor label this method 'stereoscopic 3D' and while it provides additional depth cues it may be associated with visual fatigue and discomfort. Yet other methods involve overlaying digital information on real-world objects (i.e. augmented reality) such as humans, mannequins, or physical models. In addition, holographic displays can create the illusion of 3D objects in space. While not requiring special glasses, they have a very high cost, limited resolution, and low refresh rate. A meta-analysis of the effectiveness of 3D technologies compared to all teaching methods used in anatomy education showed higher factual knowledge and significantly better results in spatial knowledge acquisition [3]. The authors also found that students showed a high degree of satisfaction and perceived the learning tools as effective. Also, Hackett and Proctor [5] conclude in their review that the majority of performed studies (74 %) indicate that 3D images in anatomical education is beneficial and student perceptions are positive. However, studies that simply confirm that students learn from using 3D images are not sufficient for informing course design or how to teach and facilitate student learning. It is important to find out how and why different features of technology-enhanced learning work. In addition, comparing a computer-based intervention to another mode of instruction, such as lectures and readings, are biased and very difficult to conduct in practice [8, 9]. While justification studies look to the past focusing on existing interventions, clarification studies that aim to understand why and how interventions work are better at supporting future developments.

### Previous research on learning and 3D images

A systematic review of evaluations of technology-enhanced learning resources by Clunie et al. [6] concluded that most studies focused on the satisfaction of students following an anatomy course or module. These studies did not, however, provide knowledge about the learning processes of students using technology and 3D images. Azer and Azer [4] conducted a review to identify studies exploring 3D anatomy models and especially reported the impact on learning. 3D anatomy models were defined as follows: "Three-dimensional (3D) anatomy models comprise digital, and non-digital (physical) models that can be moved into different positions/planes to enable the learner to learn the relationship between different anatomical structures in space and mentally manipulate objectives in three dimensions” [4, p. 81]. They conclude that research that carefully investigates different factors impacting the learning of students while using 3D anatomy models is needed. Relevant factors are related to the design of the 3D learning tool, how it is presented as well as the relationships between the curriculum and the learning environment. Studies investigating student learning concerning the use of 3D images based on pedagogical theory are quite rare. Hackett and Proctor [5] found that studies that point out learning activity design mainly refer to cognitive load theory. The mental workload associated with both 2D and 3D images was studied by Foo et al [10]. They reported the benefits of the 3D images while finding anatomical structures related to better accuracy and lower mental workload and recommended that cognitive load theory should be used to improve the curriculum design of anatomy teaching. A meta-analysis that focused on the correlation of spatial ability with anatomy assessment outcome recommended the use of cognitive load theory and the cognitive theory of multimedia learning in instructional design [11]. The authors argued that the theories can contribute to the understanding of how to design anatomy teaching for students at different levels of education and with different levels of spatial ability. In a comprehensive essay about integrating 3D visualisation technologies into undergraduate anatomy education, Keenan and Ben Awhad [12] introduce the modality appropriateness hypothesis, based on theories from the field of cognitive psychology, to inform the use of visual learning methods in anatomy education. The authors used this hypothesis to design an intervention in which they combined visualisation table-based thoracic cross-sections and digital models with a 3D-printed heart [13]. They conclude that combining multimodal learning approaches can enhance student learning and experience of cross-sectional thoracic anatomy. Silén et al [14] reported on a study that introduced 3D images based on clinical CT and MR images in curricula framed by student-centred learning theories that underpin problem-based learning. The results showed that the educational design and use of 3D images supported interest in learning and insights about biological variation and topography in contrast to arranged images of the human body.

To further understand how to facilitate and support the learning of anatomy, there is a need to know more about the student perspectives on how they can use and benefit from 3D images. This should include perspectives on the format of the 3D images as well as how learning activities are designed. Another important issue to explore is the perspective of tutors on how students use 3D images to learn anatomy. There is also a need to introduce a broader spectrum of learning theories that can improve the understanding of how to design learning activities.

### Pedagogical framework

The pedagogical framework for the present study is based on learning theories that emphasise an active, creative processing of information including cognitive, emotional, and social aspects as well as testing and practical actions [15–17]. This means that the view of learning processes is not restricted to cognitive psychology. Learning involves experiencing and using information from all senses. The lifeworld, the total sum of physical surroundings and everyday experiences that make up an individual's world, form the basis for his or her interpretations, thoughts, reactions, and actions [18]. A basic driving force or motivation to learn is a desire to understand and be able to manage situations that are perceived as relevant and meaningful [19]. Challenges, as well as confirmation and feedback, are regarded as crucial to facilitating and stimulating learning processes [20, 21]. Understanding as an outcome of learning is often referred to as meaningful learning. Such learning includes remembering facts and terminology when needed (“retention”) and the ability to make sense of the remembered facts, turning them into a meaningful whole to apply in various clinical settings [22, 23]. Meaningful learning also includes discernment of crucial elements and reorganising and combining them into comprehensible wholes [7]. The basis for the human being to discern something is variation. Variation allows us to experience differences and figure-ground structures [24]. Another important feature is that learning is fundamentally situated in a physical as well as social and cultural context affecting how learning progresses and whether and what the individual learns [25]. Moreover, learning is often a collaborative endeavour mediated by various artifacts, tools, and technologies [26]. In higher education, it is important to consider that adults have extensive experience and knowledge to build on in their learning [27]. No one enters a learning situation as a blank slate. The outcomes of learning, different kinds of knowledge, are shown in behaviour, actions, thinking, and approaches.

### Study goals

The aim of this study is to contribute to the understanding of the role of visualisation of authentic images in anatomy learning. The methodology in this study has been guided by social constructivism. This paradigm is based on the idea that knowledge is actively constructed, personal and, situated in context. Learning means doing and happens through engagement in the social world [15-17; 19]. Consequently, the focus lies in students’ actions and interactions with others and the images. Another significant point is that previous knowledge and experience will influence learning and understanding. Therefore, in this study the focus is on learning processes, not outcomes. Visualisations included in this study focus on images rendered from x-ray computed tomography (CT) and magnetic resonance (MR) of human real bodies displayed through computer-delivered technology (Sectra AB, Linköping, Sweden). Statements of students about their learning concerning their use of a visualisation table and the reflections of a tutor on the actions and expression of students will be analysed to answer the following research questions.

- How do students perceive the use and benefits of visualisation of authentic images presented on visualisation tables in relation to the process of learning anatomy?
- How does a tutor interacting with students using a visualisation table view his/her role in supporting the anatomy learning of students?

To illuminate the meaning of the experiences of students and the tutor, an interpretative qualitative approach was applied in the study [28]. As such, the focus lies on interpreting phenomena as represented by the participants. This approach acknowledges multiple realities and that meaning is constructed in interaction with other people. Researchers interpret the meaning of the data and are not considered objective. They maintain credibility by including different perspectives and applying reflexivity. Furthermore, the study was informed by Educational Design Research (EDR) as described by McKenny and Reeves [29] and Chen et al [30]. One main element in this methodology implies that the design builds on a theoretical framework relating to the actual educational practice. Another core element is that the theoretical framework is used to analyse the results.

## Methods

Students were invited to participate in specifically designed educational sessions. The study was conducted in an anatomy training unit at Karolinska Institutet, a medical university in Stockholm, Sweden, responsible for the anatomy education of several different health care professions.

### Participants

The study strove for a rich variation of backgrounds, experiences of studying anatomy, learning goals, and clinical applications [28]. To recruit participants, students from three different health care profession study programmes – medicine, physiotherapy, and nursing, were informed about the study and invited to participate. Twenty-four students accepted the invitation Table 1. The medical programme at Karolinska Institutet is five and a half years (11 semesters) long. Systematic and topographic anatomy is studied during the first three semesters. Knowledge about human anatomy is subsequently integrated and applied in different clinical courses. The courses during the first three semesters consist of lectures, human dissections, and laboratory sessions using anatomical models and a visualisation table (Sectra AB, Linköping, Sweden). The physiotherapy programme at Karolinska Institutet is three years long (6 semesters) and contains a five-week gross anatomy course during the first semester. Knowledge in anatomy is thereafter integrated and applied in practice-based and clinical courses. The anatomy course within the physiotherapy programme consists of lectures, human dissections, and laboratory sessions in small groups using anatomical models and to a lesser extent the use of a visualisation table (Sectra AB, Linköping, Sweden). The nursing programme at Karolinska Institutet is three years long (6 semesters) and starts with a course in anatomy and physiology with special reference to nursing and illness. The course in the first semester consists of lectures and laboratory sessions using anatomical models and to a small extent use of a visualisation table (Sectra AB, Linköping, Sweden). All students at the university have free access to the Visible Body Courseware (Visible Body, Newton, MA, USA), a web-based teaching and learning platform for 3D anatomy, which can be downloaded via the library at Karolinska Institutet to mobile devices such as smartphones and tablets.

**Table 1:** Descriptions of participants

Educational level	Profession	Number	Female	Male
Early part	Physiotherapy students	6	5	1
Early part	Medical students	7	4	3
Early part	Nursing students	2	2	0
Later part	Physiotherapy students	2	1	1
Later part	Medical students	6	3	3
Later part	Nursing students	1	1	0
		24		

In line with the sampling strategy described above the students in the early and later parts of their education were recruited. Students from earlier parts were studying their first to the third semester and students in the later parts were in their last two semesters of their study programme. The same tutor facilitated all sessions. The tutor was a medical student with extensive experience in teaching anatomy to medical and physiotherapy students using a visualisation table.

### Design of educational sessions

The sessions were designed based on knowledge about learning emanating from the learning theories described above. Consequently, the session was designed to allow the students to actively explore the images and the visualisation table [15–17]. Furthermore, driving forces such as meaningfulness, e.g., relation to clinical practice; challenge, e.g., self-direction and interactions with others, e.g., working in pairs, were built into the sessions [7, 18, 20, 21]. A key issue was to capture student approaches to the discernment of anatomical structures without initial help from the tutor [24, 27]. The authors represented different areas of research including medical education research, technology-enhanced learning and simulation, and anatomy education, as well as different professional backgrounds within health care.

In the sessions, a visualisation table manufactured by SECTRA (SECTRA AB, Linköping, Sweden) was used to present visualisations of the human body. The SECTRA table F18, featuring a 65 inches monitor with a 4k resolution, provided natural-sized and three-dimensional views of the anatomy of real human bodies, which were rendered CT and MR images. The table has a multi-touch screen that allowed the students to interact with the virtual body by virtually slicing, segmenting, or removing layers of tissues. Two written clinical cases were used in the sessions, one relating to the abdomen and one related to the brain. The cases were chosen based on teachers’ experience of what students usually find difficult to understand. A series of seven images, MRI and CT scans, with relevance to the clinical cases, were selected in advance by tutors in anatomy and organised for display. The images, emanated from SECTRA software, showed authentic human bodies as well as images of anatomical structures of relevance to the case. The sessions were free-standing from ordinary education and had no connection to a course-related assessment of the students. The purpose was to enable the students to approach the visualisation table without the pressure of performance related to assessment and thus allow their own thoughts about how to use this learning resource to understand the human body.

**Fig. 1:**
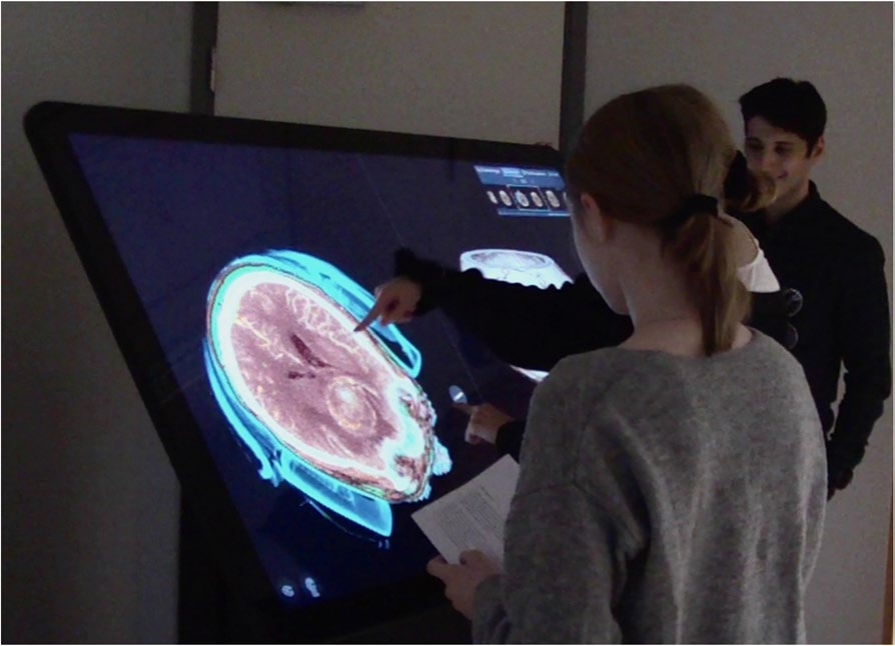
Students exploring an authentic image of the brain and the tutor as a facilitator in the back

The students worked in pairs and occasionally alone at the table exploring the selected images. A tutor with experience in using the visualisation table in ordinary anatomy education acted as a facilitator in the sessions (Fig 1). The tutor was asked to introduce the functionalities of the table, and the students were then asked to read through the written clinical cases, one at a time. The students were invited to explore the images in their own way and order and were encouraged to take a curious attitude using the functions of the table. They were instructed to think aloud about the images and their strategies to understand what they were looking at. When needed, a member of the research group prompted students with questions like “what are you looking at now” or “what are you searching for”. The purpose was to capture their thoughts and behaviour when not being instructed and guided by a tutor. The tutor was instructed to stand by to support the students if they asked for help and/or if they got stuck. At the end of each session, the tutor explained critical features of the displayed images and how they related to the written case and answered questions from the students.

### Data collection

The sessions were video and audio recorded using an ordinary video camera on a tripod and a microphone hanging from the ceiling to better capture the voices of participants. At the end of the session, the students were interviewed about the interaction with the visualisation table and their thoughts regarding different ways of learning and studying anatomy. The tutor was interviewed on two later occasions about his reflections on the session and his role as facilitator. The first was done to capture the immediate and spontaneous impression shortly after the educational session. In the second interview, the tutor was asked to reflect on selected sequences of the video recordings as stimulated recall. The selected sequences came from prior video analysis of observations of how students go about discerning anatomical structures using a visualisation table (in manuscript). The selected sequences related to identified instances in the video demonstrated the process of understanding anatomy well. They were identified and agreed upon through discussions in the research group.

### Data analysis

Qualitative content analysis [31, 32] was used to analyse the data from both the students and the tutor. The audio recordings from the sessions including the interviews with the students and the separate interviews with the tutor were transcribed verbatim. Comments from students about their learning while examining the images were noted and combined in the analysis with the interviews performed at the end of the sessions. The video recordings were included in the analysis to better understand and verify references that students made to what they had seen or done during the session. The content of the interviews was analysed both in its manifest form, i.e., participant expressions, and in its latent form, i.e., the underlying interpreted meanings [31, 32]. The transcripts were read carefully to get a sense of the whole, followed by identifying meaning units related to the participants’ descriptions of their experiences and thoughts about learning anatomy. Subsequently, the meaning units were condensed, sorted into two main categories, and finally interpreted as signifying themes representing the latent form of the findings. An example of the process is illustrated in Table 2. The analysis followed an iterative process going back and forth between parts and wholes of the video recordings, transcripts, and theoretical assumptions about learning. This process is a version of abductive analysis as presented by Timmermans and Tavory, [33]. The categories and themes were identified from the collected data but instead of setting preconceived theories aside when analysing data, the researchers let theoretical insights inform the interpretations.

**Table 2:** An example illustrating the analysis process

Condensed meaning units	Category	Theme
• you may choose for yourself • otherwise, someone else makes the choices • you get fed with what you are supposed to learn and in what way	Educational sessions using visualisation tables	Exploring on one's own is valuable

Three of the authors (CS, KK, AP) examined the video recordings and read the transcripts of each interview performed with the students and the tutor. Findings were discussed until a consensus was reached. Trustworthiness was sought by the systematic and reflexive analysis described above. All the authors contributed different perspectives and ensured increased credibility.

### Findings

Two main categories describing the accounts of learning of students and the tutor using the visualisation table were identified: 1. Interpreting 3D images and 2. Educational sessions using visualisation tables. The content of each category consists of signifying themes representing interpretations of the latent meaning of the accounts of the students and the tutor. An overview of the findings is displayed in Table 3.

**Table 3:** Overview of categories and themes in the findings

Category	Theme
Interpreting 3D images	*Realism and complexity*
	*Processes of discernment*
	*References to previous knowledge*
Educational sessions using visualisation tables	*Exploring on one's own is valuable*
	*Contextualising enhances learning experiences*
	*Combinations of learning resources are needed*
	*Working together affects the dynamics*

### Interpreting 3D images

The students discussed how they perceived the displayed images and how they went about interpreting and understanding what they saw, while the tutor reflected on his role in supporting the understanding of the students. Three themes signified their accounts: 1. Realism and complexity; 2. Processes of discernment and 3. References to previous knowledge.

### Realism and complexity

The students expressed that the images became interesting to examine, knowing that the images displayed real human beings. Images showing the anatomy of an actual person implied that the anatomy would vary between individuals and that the images did not reproduce perfect structures, as is often the case in books. In their profession in the clinic, they were expecting to meet patients with variations concerning their anatomy, and therefore it was an advantage to experience variation while also learning anatomy.

Examples of comments:

- ...and many illustrations are sort of simplified. Distances have been shortened to highlight a certain part of the nervous system and the proportions are not right at all. So, when you are trying to understand what it really looks like, it isn’t right, but here it is. This is kind of real. (Nursing student early part)
- I think it's great to see real images and how everything is not drawn perfectly with red and grey things. In working life, you won't get nice and colorful images. They will be vaguer (Medical student, early part)
- It's a kind of anatomy from the inside out. It is a real person's anatomy, not just a drawing of a person's anatomy. It's like real-life anatomy which means that you sometimes also see anatomical variations. (Medical student, later part)

Such views notwithstanding, the students also thought that realism rendered difficulties in interpreting the images. The complexity of images showing the body of real patients made it difficult to discern features in the images. All layers of tissue and organs were displayed at the same time, and unlike the distinct and colourful pictures in the anatomy book, the 3D images shifted in various shades of white, grey, and black. This made the images blurred, and it became hard to differentiate what was seen. On the one hand, the indistinctive images were challenging and motivated the students to explore the images trying to find out what they saw. On the other hand, they found it frustrating. The students argued that other learning resources, such as Visible Body (a virtual anatomy software), in which the anatomical structures are well defined, marked, and named, may be more effective at some stages of learning.

- I’m thinking of Visible Body for instance. It’s excellent in that way. You can just click and it recognizes every vessel and then you get the whole vascular system. And then you just see “that’s exactly...” (Medical students, later part)

The tutor recognised that the images that had been chosen were not always helpful in the way that he had expected and that certain images caused reactions among almost all students and affected the students’ motivation.

- The bleeding [the clinical case] was difficult, you would have to go up and down in the image to see the white spot. Many groups never checked the image very carefully and other things caught their attention.
- It [an image of the posterior abdominal wall, dissected] could have been an aha! moment but wasn’t. They had more often aha! moments around things they had no previous experience of.
- Nicer images are more fun and motivating. This [image of the vessels of the brain] was a plain X-ray but with all vessels. Everyone who looked at it was like “wow”, and they got to discover the beauty for themselves.

### Processes of discernment

An active process of searching characterised the student explanations of their attempts to interpret the 3D images. In their accounts, three aspects came to the fore: their search for different perspectives and relationships between structures, experiences of wholeness, and the need to compare images to be able to discern and understand.

The students expressed that they used the possibilities of 3D images to examine structures and organs from different angles to understand what they were looking at and how different structures are related to each other. By being able to see different layers and the depth of the body by zooming in and out, they were able to understand the three-dimensionality of the anatomical structures “in their head”. Examining images of the body from different perspectives helped students in understanding how organs relate to each other and see the sizes of organs in comparison. They stated that investigating different kinds of images of a structure from many different angles contributed to their understanding of the topographic anatomy.

- One was able to see a lot of different layers, // one was able to go deep through the person, almost. // It created a 3D image in my mind. // Zooming in and out in this way provides certainty, “ok, right, closest to the spine, it’s there, and behind the stomach..” // Because I think when you learn Visible [Body] you learn the functions rather than exactly where they [the anatomical structures] connect (Physiotherapy student later part)

Examining a human body using 3D displayed CT and MR images enabled grasping the entire body as one entity. Turning and twisting the images allowed the students to discover how organs were related to each other. The students appreciated the functions of the table that enabled them to zoom in and out and remove layers of tissue moving from the innermost parts to the surface of the skin. By following a structure centimetre by centimetre, in a structured way, the students described how they were able to discern the wholeness of the body. They found it valuable to examine organs and structures that are difficult to envision from the outside, such as the brain and vessels in their proper context in the body and doing so in detail.

- I think about wholeness. You read about the liver separately… so that you somehow get a holistic idea and not just a flat image on a piece of paper, this is how you can twist and turn, “ok, where is this placed in relation to that?” Because you realise that you’re not exactly aware of that. (Nursing student, early part)
- Here you can really, just take being able to see “ok, this is where” and then just move centimetre by centimetre and see “ok, here something is actually beginning to push through “. These kinds of things, and to see that “Okay, that’s where it starts”. It’s not just “Okay, it just is there” because we are one decimetre further down. (Physiotherapy students, early part)

An important factor was the possibility to compare images. The students described that being able to switch between images and compare an image with other images that they had viewed before helped confirm interpretations. Another kind of comparison that was considered helpful was comparing healthy, normal features with illness, or comparing the left and right sides of the body. The students explained that even if they did not understand what was wrong, the discrepancies awakened interest and made them pay attention to the anatomy.

- It’s nice to be able to compare different images to each other. You can compare someone healthy with someone who is ill and see the differences in the structures. You can distinguish things even though you don’t know what you see, you see deviations. That is what I think I can see. (Medical students, a semester 1early part)

The tutor acknowledged that when a student feels unsure of what he or she sees or is unsure if he or she is right, it can make the student hesitant and stop their way forward. A confirmation from the tutor or a peer triggered them to continue. A simple yes to verify or a cheerful nod gave the students the confirmation they needed to continue their exploration.

- They wanted to be sure before they moved on. When you confirm them they move on.

### References to previous knowledge

The students emphasised the value of previous knowledge when trying to interpret what the images showed. Recognition of an anatomical structure confirmed their hypothesis and prompted them to move on or to further investigate the images. Their references could stem from the clinical knowledge that they had gained during clinical placements. They described that, if they had come across radiological images before, they had a reference that could be useful for better understanding what they were seeing in the images displayed in the table. The students expressed a need to be able to refer to basic anatomical knowledge to make use of the 3D images. Such references could be to their prior anatomy studies, but also the simultaneous use of other learning resources, such as an atlas or internet-based learning resources. Another important source for validating their interpretation was the function and form of their own bodies.

- I identify the important structures that I actually remember clearly, some things fade away if you, as in my case, haven’t studied much anatomy since the course. You remember the most important structures; you search for those. //they work as references, and you look for recognisable references to relate everything else to (Medical student, later part)
- [it’s] important to relate to one’s own body – to experience physically (Physiotherapy student, early part)

The tutor recognised that students used different experiences and separate strategies to approach the images and the clinical case and reflected on their possible impact on learning anatomy.

- For those who were farther [in their education], it was more important to understand and reason around the case. Physiotherapy students in their first semester wanted to explore the spine even though it had nothing to do with the case.
- It's interesting how task-driven you get higher up. They have learned how important it is to focus. Which information is important for solving the case?

### Educational sessions using visualisation tables

Within the second category, students and the tutor reflected on their experiences of using the visualisation table and pedagogical setup as a resource for learning anatomy. Interpretation of the meaning of the experiences of students and the tutor and their reflections are presented in three themes: exploring on one's own is valuable; context enhances learning experiences, combinations of learning resources are needed, and working together affects the dynamics.

### Exploring on one's own is valuable

This educational session was designed to allow students to explore the visualisation table on their own. The visualisation table offered possibilities for students to use different functions such as browsing between images, zooming, slicing, removing layers, and rotating to examine the images. The students stressed their appreciation of being able to construct their own learning path and explore the images on their own and at their own pace. They described that it benefited and stimulated their learning to follow structures, find different layers, scroll, zoom in and out and choose the images they wanted to look at. Working and reasoning together with a peer while examining the images supported the learning process. It was harder to interpret the 3D images without expert help, but they expressed that they learned and remembered better by examining and discovering themselves. The students argued that having someone else telling them what they were seeing would not necessarily make it easier. They also found it more fun and stimulating to discover on their own what was displayed. When they were able to examine the images on their own, learning became more interesting.

- Student 1: It was great fun... It was fun to try finding on your own... to try to figure out what you are seeing and... Student 2: It feels like you learn a lot through speculating, just searching around and getting to see different angles. Both with bones, without bones, with contrast. You get a real 3D experience, a sense of it being a real body. It is a fun way to work. When you get to explore. Because, if all the structures are labelled then it just becomes learning by heart, this is more investigative. (Medical students, early part)
- On the other hand, since it’s... you really must try to distinguish, “ok, there must be two bones here” but when you look at pictures in books and Visible Body it’s really quick, “right, that’s red and that is blue” and then you move on. Here you must put in more effort and for me this makes me search more. (Physiotherapy student, later part)
- Also, It’s fun to navigate on your own … to find something, like we found these little.. “What are these?” So, you get interested in a different way (Physiotherapy student, early part)

The value of exploring on one’s own was also observed in the reflections of the tutor. The tutor recognised that allowing the students to explore the images on their own made the learning more pleasurable. If the students were allowed to focus on structures that they found interesting, they became more emotionally engaged and spent more time on the task. The possibility to explore on one’s own also meant that the students could choose a focus based on their needs and be able to stop and spend the due amount of time to fully understand an image or structure that they had found difficult to discern. While the students spent time on the task, this meant that they might not have enough time to cover all structures the tutor had planned for. The tutor acknowledged that students covered less of the content that would normally be the case but that students probably understood the content that they did cover better. The tutor also contended that students who were very efficient in their way of examining the images might still fail to detect important features.

- There is a risk that you miss something important if you work too target-oriented with the images. To explore is valuable to understand anatomy and to learn to analyse a problem.
- You don’t always have to tell the students, the thirst for knowledge can come from within the students.
- When the group explores on their own, they can set the focus according to their need. If the group is interested in a particular structure, they can slow down and think.

The tutor reflected on the fact that this meant taking a more passive role than would usually be the case and that stepping back as a tutor changed the way students engaged and their level of activity. By putting the students in the driver's seat, the role of the tutor turned from tutor-led lecturing to student-driven learning.

- Normally, I would have pointed out a structure and lectured about it. In this case, they had to find for themselves and reason their way to it.
- Giving the students a case that they explore on their own is better than giving them study questions because questions will direct them too much.

Another reflection from the tutor was that when the tutor decided on the focus of the lecture or what to demonstrate, this would affect the questions from the students. When the students were allowed to choose the focus for their exploration, the questions were of another kind than the tutor had expected. It might be difficult to answer all questions or to confirm something that you are unsure of as a tutor. Allowing students to explore on their own would require that the tutor has a broad competence within anatomy and can handle situations of uncertainty.

- When the students explore on their own, the questions they have are different. It requires that the tutor is broader.

### Contextualising enhances learning experiences

Written clinical cases were added to the images of real patients displayed by the visualisation table. According to the students, a connection to a clinical case contributed a helpful context and provided meaning to the images. The context that the clinical case provided was considered supportive in different ways. The students explained that receiving information about the medical history and symptoms of a real patient gave them clues about what to search for, and it became important to try to understand the problems of the patient. The students expressed that, without context, studying anatomy may simply become a matter of memorising. A context for the images created a more exciting, purposeful, and meaningful learning experience.

- Student 1 about patient cases: It became more interesting and sort of more serious, to try to discuss in the right way. To really prioritize time, right?
- Student 2: Yes, now we are going to solve this (Medical students, early part)
- I think it’s easier when you have something to relate to – such as clinical experiences – compared to just memorizing a lot of things. It has become easier now when reviewing and reiterating anatomy, why I am learning this. (Medical students, early part)

### Working together affects the dynamics

The tutor observed that working in pairs could affect the interplay between the students. Most often it resulted in more activities where students would help each other out, answering each other’s questions, confirming ideas or assumptions, collaborating while manipulating the images or using the functions of the table. In instances where one student acted as a driving force or was very dominant, the other student could sometimes take a more passive role and be quiet.

- If one student was very able the other one could be quieter and passive.
- When students worked in pairs, they were more active

### Combinations of learning resources are needed

Even though the visualisation table was assessed by the students as valuable in learning, combinations of learning resources were asked for. Students suggested that a visualisation table could be used as a complement to reading books, dissections, models and physically examining a body. The learning resources that they were using (e.g., Visible body) provided possibilities to browse, zoom and turn images in the same way as the visualisation tables but they still lacked the images of real human beings. On the other hand, they thought simplified visualisation might be needed at certain stages of learning anatomy. They suggested that 3D images in applications such as Visible body may be helpful since anatomical structures are there clearly distinguished by colours compared to CT or MR images of real bodies. Tutors facilitating learning activities were also described as important resources in combination with their own, active examination of 3D images.

- Interviewer: If you were to compare reading a book, using Visible Body and this [SECTRA] table, what are your thoughts about anatomy learning?
- Student: I think the combination is the best. Sure, here you get an image, but you still need, or I still need, to put it more into a context. Where it is, what the names are (Physiotherapy students, early part)
- Student: I think that if you manage the SECTRA table reasonably well then you could use this SECTRA machine as an introduction and avoid making mistakes and maybe improve learning, and then you get to dissect. (Medical student, early part)

## Discussion

For all professionals within health care, understanding the human body is fundamental, and learning anatomy constitutes an important part of that endeavour. The development of technology within anatomy education has triggered extensive research to establish the benefits of different innovations in anatomy education. Most studies have focused on student satisfaction and learning outcomes [6, 12, 34]. This study complements previous research by providing knowledge about how students think about how they learn and their perspectives on using technology displaying 3D images in anatomy education. In addition, reflected observations of a tutor interacting with the students added to the understanding of the perceptions of students.

This study confirms that students believe 3D images explored via advanced technology can enhance their understanding of the body. This is in accordance with findings in several reviews [1, 3–6]. The students in this study appreciated the realistic encounters with 3D images rendered from CT and MR images of human bodies. Some research on technology-enhanced learning in anatomy education has reasoned around cognitive load concerning technology-enhanced learning [5, 10, 13]. An assumption that underpins this reasoning is that reduced mental effort will improve learning and the ability to correctly identify anatomical structures. Foo et al [10] found that 3D images required less mental workload than 2D images and Ben Awadh et al [13] studied perceived challenges when interpreting 3D features of anatomical structures using a combination of 3D prints of a heart and radiological images. In contrast to this line of reasoning the students of the present study appreciated the 3D images of real patients although it complicated the interpretation of what was displayed. They did find the images indistinctive when layers of tissues covered each other, and organs were hidden behind other structures. However, it was considered exciting and instructive to get the opportunity to look inside a real human being. Structures and organs that cannot be seen at all from the outside, such as the brain and vessels, were found especially valuable to explore. The students noticed that anatomical structures do not look as perfect as they do in books. The images displayed revealed to them that anatomical structures vary between individuals in the same way they would in the clinical context. Student discovery and recognition of biological variation were also noted in a study by Silén et al [14]. These important and somewhat contradictory findings are crucial to consider. It is striking that students at all educational levels appreciated exploring images on their own without being instructed by a tutor. They appreciated being active themselves or working together with peers at their own pace. Exploring and discussing with peers was more effective for their understanding compared to having tutors point out structures, in spite the fact that they found interpreting the images difficult. Research shows that development of self-directedness in learning depends on feelings of being in charge and of having a genuine impact on the learning situation. Students need challenges as well as support and feedback in their struggle to become self-directed learners (20, 21, 27). These findings support the idea that approaches to researching anatomy education must include more than cognitive aspects of learning such as visuospatial ability or working memory.

According to the findings in this study different resources in combination seemed to make up a helpful whole compared to a single learning resource. The students emphasized the importance of combining and varying different learning resources. Using a combination of learning resources and letting technology complement traditional ways of instruction has also been argued for in other studies [12, 13, 35]. Ben Awadh et al [13] studied student learning in a design using a combination of table-based 3D images and digital models of three-dimensional printed organs which resulted in positive learning outcomes. Students talked both about parallel usage of other resources in combination with a visualisation table like dissections, books, 3D courseware, and plastinated models to prepare for and/or follow up the exploration of 3D images. Labelled and colour-coded images and structures from other anatomy software, atlas, or plastinated models helped them to understand what they were looking at. This is in resonance with Roach et al [11] who identified the importance to consider delivery modifications in anatomy learning related to students' ability to spatial reasoning. Also, Ben Awadh et al [13] found improvements in perceived challenge and performance in cross-sectional anatomy interpretation when combining the use of 3D printed models of the heart and CT images displayed as 3D images.

Although many reviews are aiming to capture the use of digital 3D images in anatomy education the teacher role has not been highlighted before. Reflected observations by the tutor in this study acknowledged that students managed well with their own discovery process. Nevertheless, they also expressed a wish for reassurance to confirm their hypothesis and interpretation of what they were looking at. It was noted that when students were not able to interpret what they saw they hesitated and stopped their exploration if they did not receive support. Finding the balance between letting students explore on their own and helping learners notice what the tutor considers important may be difficult. When guidance is appropriate and timely, it can be eye-opening and significantly help the learners move on in their exploration [36]. Here, the timing was found to be decisive. Interference by the tutor at the "wrong" moment was noticed as hampering and jeopardising the process learning for the students. The tutor highlighted that standing next to the students to support, letting the students explore on their own put certain demands on the tutors’ knowledge. Having to answer questions about unprepared topics requires the tutor to endure uncertainty.

The findings of the present study indicate that there are many factors to consider when designing supportive and effective anatomy education. The tutor role, the use of different learning resources, the choice of images, and student interactions were found to affect students learning. Statements from the students and the tutor underline that the use of 3D images in anatomy education involves a complex learning process and designing appropriate anatomy education thus requires attention to a wide range of factors. In a review by Freeman et al [37] active learning approaches such as group problem-solving and studio or workshop course designs in anatomy and physiology courses, were found to increase student performance in examinations. According to the students in this study, the functionalities and interaction with the table were useful for their learning. The SECTRA table has been developed to provide these possibilities [12, 38] and the students confirm its value. The table allowed them to be active in their searching processes by zooming, scrolling, browsing between, and comparing images to discern and interpret anatomical structures. The functions of the visualisation table enabled a deeper understanding of the topography of anatomical structures including their sizes, spatial relationships, and relations to the whole body. Context came to the fore as a meaningful factor making it possible to connect what the students saw to something familiar. This context provided them a reason why they should try to interpret the images. CT and MR images from real patients related to a written clinical case stimulated the interest to interpret and connect to a future profession. This was stated by students from all educational levels. Even if students at early stages had difficulties understanding the disease and pathophysiology of a patient, it was still stimulating, and students at later stages received confirmation and feedback on their knowledge. However, the student's previous knowledge of both anatomy, related subjects such as physiology and pathology, as well as experiences with CT and MR images, were highlighted as important for interpreting the images. Previous knowledge was used as a reference when trying to discern anatomical structures. The educational levels of students and their clinical experiences are, not surprisingly, crucial for managing the interpretation of 3D images. In addition, the students’ notion of their future professions seemed to matter regarding their interest in different anatomical structures, their motivation to find out what they saw and about the possible diseases of the patients. This was commented on by the tutor who observed that students on different educational levels used different strategies to examine the images.

To increase the possibilities of transferring the empirical findings of this study to other contexts, the findings have been situated within theories of learning. The theoretical framework recognises learning as the processing of information cognitively, emotionally, socially, and in practical actions. Motivation is regarded as a basic driving force for learning. If motivation is primarily built on external stimulation, such as passing a test or pleasing someone else, it risks being fragile and of short duration. Far more effective in the long run are motivational factors that build on a feeling of mastery and meaningful experiences [19, 39]. Findings in this study reveal several such motivational factors. Students expressed a felt joy when allowed to master their own learning process. The role of the tutor seemed important in supporting these conditions. The way the educational intervention was designed allowed students to take their own initiatives and get a sense of ownership of their learning process. When they succeeded in their ambitions, they received a sense of mastery and competence [21, 39, 40]. The students expressed interest and excitement about studying images of real bodies, even if 3D displayed CT and MR images were difficult to interpret. They appreciated the sense of looking at and exploring the anatomy of a real human being with natural variations and the possibility to grasp the wholeness. The challenge of realism and complexity was stimulating and promoted further interest to study anatomy. According to variation theory, a fundament in learning includes experiences of variety to be able to discern crucial elements and reorganise and combine them into comprehensible wholes [19, 24]. Variation theory may explain the positive outcomes of using a modality appropriateness hypothesis [12, 13]. The students in this study emphasised not only the value of variation related to different 3D modalities but also a need for variation connected to other kinds of learning resources. Possibilities to make connections with the 3D images and the future professions of the students were also mentioned as meaningful. Creating and/or offering a context for learning experiences is well known to be both motivational and of importance for application and transfer to other contexts [27]. To recognise that learning depends on cognitive, emotional, and social processes as well as practical actions involves considering the meaning of context in educational design [15–17]. Another cornerstone in learning is knowledge about the need to actively process information, use all senses, and be able to construct comprehension and understanding [15, 17, 23]. The students in this study stated that their own exploration of images was valuable in their learning process. The tutor acknowledged the advantages of the discovery processes of the students and noticed that they were able to progress in their interpretations on their own. The students also called for different kinds of support in their exploration. Acknowledging the statements of the students, the tutor highlighted the difficult balance between supporting or becoming instructive in the students’ learning processes [20]. The experiences of both students and the tutor resonates with assumptions about learning in line with student-centred learning [16]. Several educational models emphasising student-centred learning, such as Problem based-learning and Team-based learning, stress the importance of students' own inquiry processes and self-directed learning. Fundamental assumptions declare that if students become interested in what they really believe they need to know, that which searches for answers meaningful, the likelihood of remembering and comprehending what they study will increase [15, 19, 22].

### Limitations

According to variation sampling in qualitative research, the participants reflected diversity in terms of profession, the experience of anatomy education, and sex. Nevertheless, the number of students is limited in the study and other perspectives would probably appear if more and other students had been recruited. Especially, a higher number of nursing students on different levels would have strengthened the study. Only one tutor was asked to participate. This is a limitation of possible tutor perspectives. The choice was made to ensure that the same tutor participated in all sessions and met all students. This enabled in-depth reflection on all the students' expressions and behaviour. The themes represent interpretations of the accounts of the whole student group. At an individual level, however, there might be different perceptions about how to learn anatomy. The spatial ability of the participants was not considered in this study. Therefore, it is not known how the spatial ability of the participants may have affected the findings. This is a limitation since the spatial ability is important when learning using 3D visualisations [11]. Another limitation might be the process of analysis and interpretation. Three of the authors attended the educational sessions and were also responsible for the interviews and analysis. None of these authors were experts in anatomy which may have limited interpretations of the data. To achieve credibility, peer discussions of the findings were employed by consulting all authors. The educational design and theoretical framework have been thoroughly described to enable review and possibilities to apply the findings in different kinds of educational contexts.

## Conclusion

Learning is a complex process. Both internal factors related to the student, and external factors related to the physical and social environment will impact the process and outcome of learning. This makes it difficult to measure learning and make simple generalisations about cause-and-effect relationships. Nevertheless, this study reveals several important factors to be considered when trying to design rewarding educational sessions using 3D images in anatomy education. Such factors include the combination of resources for learning, the use of complex authentic images and cases, allowing students to explore on their own while balancing tutor guidance and providing an opportunity for students to compare complex images with clearer images. This study shows that students think that authentic 3D images are of help in anatomy learning. Authenticity is valuable in students' learning processes since it triggers interest in possible connections to real patients and future professional clinical reasoning. In addition, it highlights anatomical variations. The complexity of authentic images stimulates the interest in exploring what is shown but they can also become obstacles and diminish the desire to explore further. Visualisation combined with possibilities for zooming, slicing, turning, and comparing images support their learning when trying to understand the topography of anatomical structures including their sizes, spatial relationships, and the relations to the whole body. Offering possibilities for students to master their own exploration of 3D images seems crucial to creating meaningfulness in their learning process. Reflected observations by the tutor confirmed that students to a certain degree were successful on their own. However, the tutor also acknowledged the students' need for help. The clue seems to be to find a balance between own discovery and guidance from a tutor and other resources. Facilitating balanced educational sessions requires a knowledgeable tutor both concerning anatomy and learning processes. The students also stressed a need to compare the complex 3D images to clear images showing segregated layers and labelled structures. The future professions of the students, their educational levels, experiences of looking at CT and MR images, and familiarity with technology all matter for the benefit of learning anatomy using authentic 3D images. We would suggest that 3D images can be used within all health education programmes at all levels in complement to other resources for learning anatomy. Questions of when and how to best use authentic 3D images in a certain course require careful consideration.

## Data Availability

The datasets generated and analysed during the current study are not publicly available due to ethical reasons connected to the students' informed consent. The data generated during this study consist of audio and video recordings of participants who have been assured anonymity. On reasonable request, data can be made available from the corresponding author.
